# Multisensor Parallel Largest Ellipsoid Distributed Data Fusion with Unknown Cross-Covariances

**DOI:** 10.3390/s17071526

**Published:** 2017-06-29

**Authors:** Baoyu Liu, Xingqun Zhan, Zheng H. Zhu

**Affiliations:** 1 School of Aeronautics and Astronautics, Shanghai Jiao Tong University, 800 Dongchuan Road, Minhang District, Shanghai 200240, China; abao-liu@163.com; 2 Department of Mechanical Engineering, York University, 4700 Keele Street, Toronto, ON M3J 1P3, Canada; gzhu@yorku.ca

**Keywords:** largest ellipsoid, distributed data fusion, parallel structure, unknown cross-covariances, multisensor

## Abstract

As the largest ellipsoid (LE) data fusion algorithm can only be applied to two-sensor system, in this contribution, parallel fusion structure is proposed to introduce the LE algorithm into a multisensor system with unknown cross-covariances, and three parallel fusion structures based on different estimate pairing methods are presented and analyzed. In order to assess the influence of fusion structure on fusion performance, two fusion performance assessment parameters are defined as Fusion Distance and Fusion Index. Moreover, the formula for calculating the upper bounds of actual fused error covariances of the presented multisensor LE fusers is also provided. Demonstrated with simulation examples, the Fusion Index indicates fuser’s actual fused accuracy and its sensitivity to the sensor orders, as well as its robustness to the accuracy of newly added sensors. Compared to the LE fuser with sequential structure, the LE fusers with proposed parallel structures not only significantly improve their properties in these aspects, but also embrace better performances in consistency and computation efficiency. The presented multisensor LE fusers generally have better accuracies than covariance intersection (CI) fusion algorithm and are consistent when the local estimates are weakly correlated.

## 1. Introduction

Multiple sensors have been widely employed in various systems, such as the integrated navigation system of driverless cars. Multisensor data fusion aims to achieve an accurate, robust and reliable representative of the target of interest by combining the information from different used sensors. The data (estimate) fusion algorithms of multisensor system can be generally classified to centralized fusion algorithms and distributed fusion algorithms. The centralized fusion algorithms can obtain the globally optimal estimate by directly combining sensor outputs to an augmented measurement. However, such fusion architecture leads to a heavy computational burden; furthermore, the fused estimate will be easily corrupted if any sensor degenerates. The distributed fusion algorithms can reduce the computational burden and facilitate fault detection or isolation more conveniently through combining the local estimates from sensors by weighting matrices. In the distributed data fusion of multisensor system, once the cross-covariances among local estimates are known exactly, globally optimal or suboptimal estimates can be obtained by using optimal fusion algorithms, such as the two-sensor Bar-Shalom and Campo (BC) algorithm [1] or its version for multisensor systems [2] in the sense of maximum likelihood (ML), or the optimal distributed Kalman fuser weighted by matrices [3] in the sense of linear unbiased minimum variance (LUMV). However, in many applications, these cross-covariances are difficult to be computed accurately; one critical issue of multisensor data fusion in the case is how to merge the local estimates efficiently to achieve a fused estimate that has high accuracy and simultaneously is consistent. With this aim, various distributed data fusion algorithms for multisensor systems with unknown cross-covariances are proposed, such as the convex combination (CC) algorithm [4], ellipsoidal intersection (EI) algorithm [5], largest ellipsoid (LE) algorithm [6], covariance intersection (CI) algorithm [7] and their variants the internal ellipsoidal approximation (IEA) algorithm [8], fast covariance intersection (FCI) algorithm [9], etc. The CI algorithm is of special concern and has been widely applied to many fields, for it yields a common upper bound of actual fused error covariance regardless of unknown cross-covariances. When all the local estimates are consistent, the CI algorithm gives a consistent fused estimate with higher accuracy than each local estimate. However, the CI algorithm is based on the optimization of a multi-dimensional nonlinear cost function, which results in a large computational burden; in addition, it overestimates the actual fused error covariance and purses consistency at the expense of accuracy, which leads to a significant decrease in performance [8,10,11]. Although several improvements [12,13,14,15,16,17] have been developed for the CI algorithm since it was proposed, these drawbacks have not yet been essentially resolved. Compared to the CI algorithm, the CC algorithm and the EI algorithm, the LE algorithm does not need any optimizations of cost functions, but they may become inconsistent in some cases owing to the unknown cross-covariances.

The LE algorithm is a two-sensor fusion algorithm that obtains a new estimate from two local estimates based on a series of matrix transformations. Instead of computing a tightest fused error covariance ellipsoid which encloses the intersection region of the covariance ellipsoids of local estimates in the CI algorithm, the LE algorithm computes the largest ellipsoid contained within that intersection region, which leads to a tighter fused error covariance ellipsoid. Besides not requiring optimization of a cost function, the LE algorithm has many other advantages. It comparatively has better consistency performance than the CC algorithm and has better actual fused accuracy than the EI algorithm and CI algorithm in general. The two shortcomings of the LE algorithm are that the LE algorithm is limited to two-sensor applications and its consistency can not be unconditionally satisfied for correlated local estimates. However, although the cross-covariances are hard to be known exactly, some information about the dependency properties among local estimates might be possible to be obtained for users, such as the correlation level [11]. When the local estimates are weakly correlated, the adverse impact of the correlation on fusion consistency performance will be limited. On the other hand, although the performance of a fuser is basically determined by its fusion algorithm, the fusion structure also has an important influence on it . A sequential covariance intersection (SCI) Kalman filter is proposed by applying sequential processing to reduce the complexity and computational burden of the batch CI algorithm [18]. A two-level fusion structure is presented, which combines the merits of the measurement fusion algorithm and CI algorithm to reduce calculation burden and get a more accurate fused estimate [19]. In addition, Kalman-Particle filtering with a cascaded structure is conducted to reduce the complexity of a high dimensional state space model, which leds to an easier tuning and more precise debugging, as well as reduced computation time [20]. Therefore, when the local estimates are not strongly correlated, how to extend the LE algorithm to multisensor cases with proper fusion structure and simultaneously improve its performances in accuracy and consistency is worth being addressed.

This paper proposes a largest ellipsoid fusion Kalman filtering with parallel fusion structure for the data fusion of multisensor system with unknown cross-covariances among local estimates, which realizes the multisensor fusion as a tree form with each level consisting of one or a series of parallel LE fusions. With parallel fusion structure, the data processing task of the proposed filtering is amenable to multiprocessor implementation. Three different estimate pairing methods for constructing the parallel fusion structure are given, and two fusion performance assessment parameters of Fusion Distance and Fusion Index for assessing the influence of fusion structure on fusion performance are defined. The attributes of the presented fusers in Fusion Distance, Fusion Index, and accuracy relation based on covariance ellipsoid, as well as the formula for calculating the upper bounds of actual fused error covariances of the presented fusers regardless of unknown cross-covariances, are provided. In order to verify the effectiveness of the proposed filtering, simulation examples are carried out to compare the performances of CI algorithm, optimal distributed Kalman fuser weighted by matrices, LE fuser with sequential structure and the LE fusers with the proposed parallel structures.

## 2. Preliminaries

An estimate of stochastic state $x\in R^{n\times 1}$ usually can be characterized with a Gaussian distribution ${\hat{x}}_{e}∼N\left({\bar{\hat{x}}}_{e},P_{e}\right)$, where ${\bar{\hat{x}}}_{e}\in R^{n\times 1}$ and $P_{e}\in R^{n\times n}$, respectively, represent the mean and fused error covariance. The estimate is said to be consistent (or conservative) only when its actual fused error covariance ${\bar{P}}_{e}=E\left[\left({\hat{x}}_{e}-x\right){\left({\hat{x}}_{e}-x\right)}^{T}\right]$ satisfies ${\bar{P}}_{e}\leq P_{e}$ [12,13], the superscript T denotes the transpose, and the notation E(∗) denotes the expectation. The fused error covariance represents the fused accuracy, and the actual fused error covariance indicates the actual fused accuracy. Given real symmetric positive definite matrices $P_{a}\in R^{n\times n}$ and $P_{b}\in R^{n\times n}$, $P_{a}\geq P_{b}$ denotes $P_{a}-P_{b}$ as positive semi-definite. Then, $\mathrm{tr}\left(P_{a}\right)\geq \mathrm{tr}\left(P_{b}\right)$, $P_{a}^{-1}\leq P_{b}^{-1}$ and $CP_{a}C^{T}\geq CP_{b}C^{T}$ hold for any row full rank matrix C [21], the superscript −1 denotes the inverse, and the notation tr(∗) denotes the trace. The estimate ${\hat{x}}_{e}$ with Gaussian distribution also can be illustrated by multi-dimensional covariance ellipsoid whose contour of one sigma is defined by ${ℜ}_{\left({\bar{\hat{x}}}_{e},P_{e}\right)}\equiv \left\{x|{\left(x-{\bar{\hat{x}}}_{e}\right)}^{T}P_{e}^{-1}\left(x-{\bar{\hat{x}}}_{e}\right)=1\right\}\;$. The center of ${ℜ}_{\left({\bar{\hat{x}}}_{e},P_{e}\right)}$ is ${\bar{\hat{x}}}_{e}$, and the lengths of the semi-axes of ${ℜ}_{\left({\bar{\hat{x}}}_{e},P_{e}\right)}$ are given by $\sqrt{\sigma_{i}}$, where $\sigma_{i}$ are the singular values of the matrix $P_{e}$. Hence, larger covariance ellipsoid means worse accuracy. For two estimates ${\hat{x}}_{a}∼N\left({\bar{\hat{x}}}_{a},P_{a}\right)$ and ${\hat{x}}_{b}∼N\left({\bar{\hat{x}}}_{b},P_{b}\right)\;$, the necessary and sufficient condition for $P_{a}\geq P_{b}$ is ${ℜ}_{\left({\bar{\hat{x}}}_{a},P_{a}\right)}⊃{ℜ}_{\left({\bar{\hat{x}}}_{b},P_{b}\right)}$, which means that the ellipsoid ${ℜ}_{\left({\bar{\hat{x}}}_{a},P_{a}\right)}$ encloses the ellipsoid ${ℜ}_{\left({\bar{\hat{x}}}_{b},P_{b}\right)}$.

Consider the discrete time-invariant linear stochastic system with multiple sensors

(1) $$\begin{array}{l} x\left(t+1\right)=\Phi x\left(t\right)+\Gamma w\left(t\right)\\ y_{i}\left(t\right)=H_{i}x\left(t\right)+v_{i}\left(t\right),\;\;i=1,2,\ldots ,L \end{array},$$

where t is the discrete time, L is the number of sensors, $x\left(t\right)\in R^{n\times 1}$ is the state, $y_{i}\left(t\right)\in R^{m\times 1}$ is the measurement, w(t) and $v_{i}\left(t\right)\in R^{m\times 1}$ are the uncorrelated white noises with zero mean and covariance matrices Q and $R_{i}$, respectively; Φ, Γ, $H_{i}$ are constant matrices with compatible dimensions, and (Φ,Γ) is a completely controllable pair, $\left(\Phi ,H_{i}\right)$ is a completely observable pair. The subsystem based on the ith sensor of multisensor system (1) has local steady-state Kalman filter as [22]

(2) $${\hat{x}}_{i}\left(t|t\right)=\left(I_{n}-K_{i}H_{i}\right)\Phi {\hat{x}}_{i}\left(t-1|t-1\right)+K_{i}y_{i}\left(t\right),$$

with $K_{i}=\Sigma_{i}H_{i}^{T}{\left(H_{i}\Sigma_{i}H_{i}^{T}+R_{i}\right)}^{-1}$, where $I_{n}$ denotes the n×n unit matrix, $\Sigma_{i}$ satisfies the Riccati equation

(3) $$\Sigma_{i}=\Phi \left[\Sigma_{i}-\Sigma_{i}H_{i}^{T}{\left(H_{i}\Sigma_{i}H_{i}^{T}+R_{i}\right)}^{-1}H_{i}\Sigma_{i}\right]\Phi^{T}+\Gamma Q\Gamma^{T}.$$

The local filtering error covariance is given by

(4) $$P_{i}=P_{ii}=\left(I_{n}-K_{i}H_{i}\right)\Sigma_{i},$$

and the local filtering error cross-covariance between the subsystems of the ith and jth sensor satisfies the Lyapunov equation

(5) $$P_{ij}=\left(I_{n}-K_{i}H_{i}\right)\Phi P_{ij}{\left[\left(I_{n}-K_{j}H_{j}\right)\Phi \right]}^{T}+\left(I_{n}-K_{i}H_{i}\right)\Gamma Q\Gamma^{T}{\left(I_{n}-K_{j}H_{j}\right)}^{T},$$

Then, the overall error covariance of the multisensor system is $\Sigma =\left(P_{ij}\right)\in R^{Ln\times Ln},\;i,j=1,2,\ldots ,L$.

## 3. Distributed Fusion Algorithms

For unbiased state estimation, both the state and its error covariance should be estimated.

### 3.1. Optimal Distributed Kalman Fuser Weighted by Matrices

Once all of the local filtering error covariances and cross-covariances are obtained, the optimal distributed Kalman fuser weighted by matrices under LUMV for multisensor system (1) is given by [3]

(6) $${\hat{x}}_{\left(L\right)}^{O}=\sum_{i=1}^{L}A_{i}^{O}{\hat{x}}_{i}\left(t|t\right),$$

where $A_{i}^{O}$ is the optimal state estimation weighting matrix corresponding to the ith local estimate and computed by

(7) $$\left[A_{1}^{O},A_{2}^{O},\ldots ,A_{L}^{O}\right]={\left(e^{T}\Sigma^{-1}e\right)}^{-1}e^{T}\Sigma^{-1},$$

where $e={\left[I_{n},I_{n},\ldots ,I_{n}\right]}^{T}$ is a Ln×n matrix. The error covariance of ${\hat{x}}_{\left(L\right)}^{O}$ is given as

(8) $$P_{\left(L\right)}^{O}=\left[A_{1}^{O},A_{2}^{O},\ldots ,A_{L}^{O}\right]\Sigma {\left[A_{1}^{O},A_{2}^{O},\ldots ,A_{L}^{O}\right]}^{T}={\left(e^{T}\Sigma^{-1}e\right)}^{-1},$$

with the accuracy relation $P_{\left(L\right)}^{O}\leq P_{i},\;i=1,2,\ldots ,L$. As shown from (6)–(8), it is necessary that all the covariances $P_{ij},\;\;\;i,j=1,2,\ldots ,L$ should be exactly known in the calculation of ${\hat{x}}_{\left(L\right)}^{O}$ and $P_{\left(L\right)}^{O}$; however, such a condition can not be satisfied in many applications. Moreover, $\Sigma^{-1}$ is also required to be computed, which will result in heavy computational burden when the number of sensors is large.

### 3.2. Largest Ellipsoid Fusion Algorithm

The LE algorithm obtains a new estimate from two local estimates based on a series of matrix transformations. Given two local estimates ${\hat{x}}_{1}∼N\left({\bar{\hat{x}}}_{1},P_{1}\right)$ and ${\hat{x}}_{2}∼N\left({\bar{\hat{x}}}_{2},P_{2}\right)$. Firstly, we diagonalize $P_{1}$ as

(9) $$U^{T}P_{1}U=W=\mathrm{diag}\left(\lambda_{1},\lambda_{2},\ldots ,\lambda_{n}\right),$$

where the notation diag(∗) denotes forming a diagonal matrix sequentially using the elements in parentheses, and U is an orthogonal matrix. Then, we execute the following transformations:

(10) $$\begin{array}{l} W^{-\frac{1}{2}}U^{T}P_{1}UW^{-\frac{1}{2}}=P_{1}^{'}=I_{n}\\ W^{-\frac{1}{2}}U^{T}P_{2}UW^{-\frac{1}{2}}=P_{2}^{'} \end{array},$$

where $W^{-\frac{1}{2}}={\left[\mathrm{diag}\left(\sqrt{\lambda_{1}},\sqrt{\lambda_{2}},\ldots ,\sqrt{\lambda_{n}}\right)\right]}^{-1}$. Applying a second diagonalization, we have

(11) $$\begin{array}{l} V^{T}P_{1}^{'}V=P_{1}^{*}=I_{n}\\ V^{T}P_{2}^{'}V=P_{2}^{*}=\mathrm{diag}\left(\lambda_{1}^{*},\lambda_{2}^{*},\ldots ,\lambda_{n}^{*}\right) \end{array},$$

where V is an orthogonal matrix. Then, we define

(12) $$\begin{array}{l} {\hat{x}}_{1}^{*}=V^{T}W^{-\frac{1}{2}}U^{T}{\hat{x}}_{1}\\ {\hat{x}}_{2}^{*}=V^{T}W^{-\frac{1}{2}}U^{T}{\hat{x}}_{2} \end{array}.$$

After these transformations, we obtain two new estimates in the new Euclidean space $R^{*}$ as ${\hat{x}}_{1}^{*}$ and ${\hat{x}}_{2}^{*}$ with error covariances $P_{1}^{*}$ and $P_{2}^{*}$, respectively. From (11), we know that both $P_{1}^{*}$ and $P_{2}^{*}$ are diagonal matrices. The fused estimate of LE algorithm in Euclidean space $R^{*}$ is given by

(13) $${\hat{x}}_{LE}^{*}={\left[{\left(P_{1}^{*}\right)}^{-1}+{\left(P_{2}^{*}\right)}^{-1}\right]}^{-1}\left[{\left(P_{1}^{*}\right)}^{-1}{\hat{x}}_{1}^{*}+{\left(P_{2}^{*}\right)}^{-1}{\hat{x}}_{2}^{*}\right],$$

with fused error covariance

(14) $$P_{LE}^{*}=SP_{1}^{*}+\left(I_{n}-S\right)P_{2}^{*},$$

where $S=\mathrm{diag}\left(s_{1},s_{2},\ldots ,s_{n}\right)$ with

(15) $$s_{i}=\{\begin{array}{l} 1,\;\lambda_{i}^{*}\geq 1\\ 0,\;else\; \end{array}.$$

The fused estimate of LE algorithm in the original Euclidean space R is obtained by

(16) $$\;{\hat{x}}_{L}^{E}=UW^{\frac{1}{2}}V{\hat{x}}_{LE}^{*}=A_{1}^{LE}{\hat{x}}_{1}+A_{2}^{LE}{\hat{x}}_{2},$$

with fused error covariance

(17) $$P^{LE}=UW^{\frac{1}{2}}VP_{LE}^{*}V^{T}W^{\frac{1}{2}}U^{T}=B_{1}^{LE}P_{1}+B_{2}^{LE}P_{2},$$

where the weighting matrices are calculated as follows:

(18) $$\begin{array}{l} A_{1}^{LE}=UW^{\frac{1}{2}}V{\left[{\left(P_{1}^{*}\right)}^{-1}+{\left(P_{2}^{*}\right)}^{-1}\right]}^{-1}{\left(P_{1}^{*}\right)}^{-1}V^{T}W^{-\frac{1}{2}}U^{T}\\ A_{2}^{LE}=UW^{\frac{1}{2}}V{\left[{\left(P_{1}^{*}\right)}^{-1}+{\left(P_{2}^{*}\right)}^{-1}\right]}^{-1}{\left(P_{2}^{*}\right)}^{-1}V^{T}W^{-\frac{1}{2}}U^{T} \end{array},$$

(19) $$\begin{array}{l} B_{1}^{LE}=UW^{\frac{1}{2}}VSV^{T}W^{-\frac{1}{2}}U^{T}\\ B_{2}^{LE}=UW^{\frac{1}{2}}V\left(I_{n}-S\right)V^{T}W^{-\frac{1}{2}}U^{T} \end{array}.$$

From (16)–(19), we know that both the fused state and fused error covariance of LE algorithm are the linear estimates. The covariance ellipsoid of $P^{LE}$ is the largest one contained within the intersection region ${ℜ}_{\left({\bar{\hat{x}}}_{1},P_{1}\right)}\cap {ℜ}_{\left({\bar{\hat{x}}}_{2},P_{2}\right)}$, and it is obvious that $P^{LE}\leq P_{1}$, $P^{LE}\leq P_{2}$. For any two unbiased local estimates of state x with Gaussian distributions, because both the optimal distributed Kalman fuser estimate ${\hat{x}}_{\left(2\right)}^{O}$ and the LE algorithm estimate ${\hat{x}}_{L}^{E}$ are linear unbiased estimates of x, and ${\hat{x}}_{\left(2\right)}^{O}$ is the LUMV estimate, we have $P_{\left(2\right)}^{O}\leq {\bar{P}}^{LE}$, where ${\bar{P}}^{LE}=E\left[\left({\hat{x}}^{LE}-x\right){\left({\hat{x}}^{LE}-x\right)}^{T}\right]$ is the actual fused error covariance of the LE algorithm. Furthermore, it can be easily proven that ${\bar{P}}^{LE}<P^{LE}$ when $P_{1}$ and $P_{2}$ are independent.

**Figure 1:**
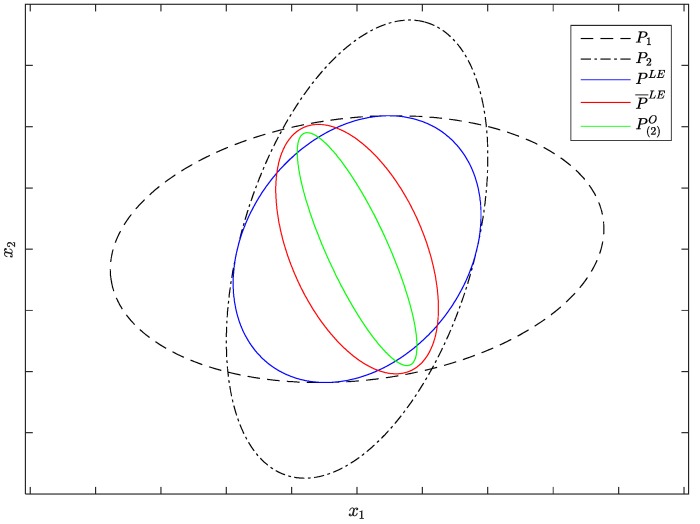
An accuracy relation example of the (largest ellipsoid) LE algorithm.

There are mainly two drawbacks for the LE algorithm. The first one is that the LE algorithm can only handle two sensors at a time. The second one is that the LE algorithm can’t guarantee its consistency, which implies that ${\bar{P}}^{LE}\leq P^{LE}$ will be unsatisfied in some cases. In the two-sensor case, according to [23], for any point $x\in {ℜ}_{\left({\bar{\hat{x}}}_{1},P_{1}\right)}\cap {ℜ}_{\left({\bar{\hat{x}}}_{2},P_{2}\right)}$, there is a feasible cross-covariance $P_{12}$ that lets $x\in P_{\left(2\right)}^{O}$. As mentioned above, the covariance ellipsoid of $P^{LE}$ is the largest one contained within the intersection region ${ℜ}_{\left({\bar{\hat{x}}}_{1},P_{1}\right)}\cap {ℜ}_{\left({\bar{\hat{x}}}_{2},P_{2}\right)}$, but it generally doesn’t cover the whole intersection region. Combining the accuracy relation between the optimal distributed Kalman fuser and LE algorithm, if the $P_{12}$ lets $P_{\left(2\right)}^{O}≰P^{LE}$, ${\bar{P}}^{LE}\leq P^{LE}$ will be unsatisfied. A two-dimensional example of a situation like that is shown in Figure 1. In Figure 1, the *x*-axis and the *y*-axis represent the first and second dimension of the state, respectively; the covariance ellipse of $P^{LE}$ is the largest ellipse contained within the intersection region ${ℜ}_{\left({\bar{\hat{x}}}_{1},P_{1}\right)}\cap {ℜ}_{\left({\bar{\hat{x}}}_{2},P_{2}\right)}$, but ${\bar{P}}^{LE}≰P^{LE}$, which implies that the fused result is inconsistent.

However, the covariance ellipsoid corresponding to the actual fused error covariance accounting for the dependence of local estimates will become smaller as the dependence becomes weaker [1], which means that the LE algorithm is likely to be consistent when the local estimates are weakly correlated. On the other hand, by comparing (16)–(19) to (6) and (8), we find that, unlike the optimal distributed Kalman fuser, which computes its state and error covariance using the same weighting matrices, the error covariance estimation of LE algorithm is independent of its state estimation. If the LE algorithm can be extended to the multisensor system, it is possible to raise its actual fused accuracy and subsequently improve its consistency performance by taking full advantage of the information of each sensor.

## 4. Multisensor Largest Ellipsoid Fusers

### 4.1. Multisensor Largest Ellipsoid Fuser with Sequential Structure

**Figure 2:**
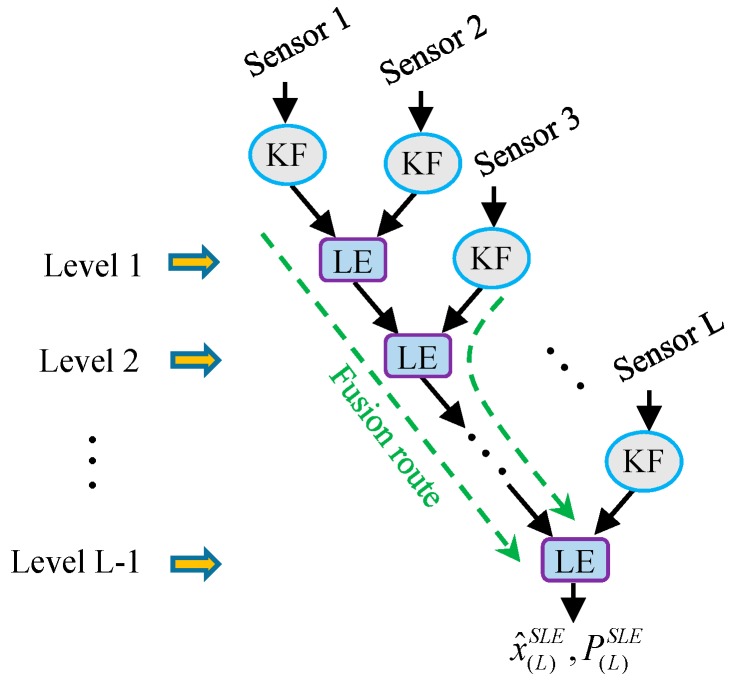
The structure of the SLE fuser.

One way to extend the application of LE algorithm from a two-sensor case to a multisensor case is applying the sequential processing method as the SCI algorithm proposed in [18]; here, we label such multisensor LE fuser as a Sequential Largest Ellipsoid (SLE) fuser. By doing so, the multisensor LE fusion for multisensor system (1) consists of L−1 sequential LE fusions. The structure of SLE fuser is shown schematically in Figure 2. In Figure 2, ‘KF’ represents the local steady-state Kalman filter of each subsystem, ‘LE’ represents a LE algorithm operation, and the green dashed lines or curves indicate the routes via which the local estimates are fused into the final fused result.

The SLE fuser has a recursive sequential form as

(20) $$\begin{array}{c} {\hat{x}}_{i+1}^{SLE}=A_{1/i}^{LE}{\hat{x}}_{i}^{SLE}+A_{2/i}^{LE}{\hat{x}}_{i+1}\\ P_{i+1}^{SLE}=B_{1/i}^{LE}P_{i}^{SLE}+B_{2/i}^{LE}P_{i+1}\; \end{array},\;i=1,2,\ldots ,L-1.$$

with initial values $P_{1}^{SLE}=P_{1}$, $x_{1}^{SLE}={\hat{x}}_{1}$; $A_{1/i}^{LE}$ and $A_{2/i}^{LE}$ are the weighting matrices for state estimation corresponding to the LE fusion in the ith fusion level; $B_{1/i}^{LE}$ and $B_{2/i}^{LE}$ are the weighting matrices for error covariance estimation corresponding to the LE fusion in the ith fusion level. In addition, the fused estimate of SLE fuser is defined as

(21) $$\begin{array}{l} {\hat{x}}_{\left(L\right)}^{SLE}={\hat{x}}_{L}^{SLE}\\ P_{\left(L\right)}^{SLE}=P_{L}^{SLE} \end{array}.$$

Combining (20) with (21) to expand the recursive sequential form of the SLE fuser yields linear expressions as follows:

(22) $$\begin{array}{l} {\hat{x}}_{\left(L\right)}^{SLE}=\sum_{i=1}^{L}A_{i}^{SLE}{\hat{x}}_{i}\\ P_{\left(L\right)}^{SLE}=\sum_{i=1}^{L}B_{i}^{SLE}P_{i} \end{array},$$

where the weighting matrices are computed by

(23) $$\begin{array}{c} A_{i}^{SLE}=A_{2/i-1}^{LE}\prod_{j=i}^{L-1}A_{1/j}^{LE}\;\;\;\\ B_{i}^{SLE}=B_{2/i-1}^{LE}\prod_{j=i}^{L-1}B_{1/j}^{LE}\;\;\;\; \end{array},$$

with $A_{2/0}^{LE}=B_{2/0}^{LE}=I_{n}$. From (23), we know that both $A_{i}^{SLE}$ and $B_{i}^{SLE}$ are the multiplication results of all the LE fusion weighting matrices that the ith local estimate encounters in its fusion route.

### 4.2. Multisensor Largest Ellipsoid Fusers with Parallel Structures

**Figure 3:**
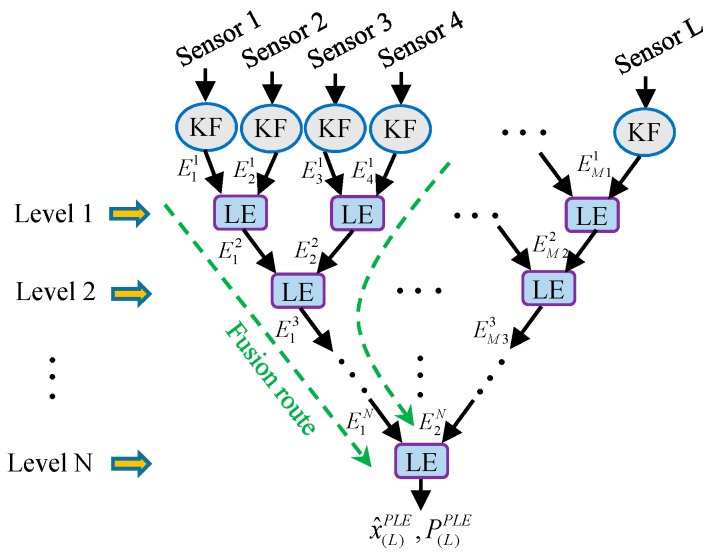
The structure of the PLE fuser.

As we can see from Figure 2, the sensors must be fused sequentially in the SLE fuser, which makes the SLE fuser inefficient in multiprocessor operations; in addition, the studies in the following part of this paper will show that the performance of SLE fuser is relatively poor. To handle such disadvantages, a largest ellipsoid fusion Kalman filtering with parallel structure for the data fusion of multisensor system, called a Parallel Largest Ellipsoid (PLE) fuser, is also proposed in this work. It realizes the date fusion of multisensor system (1) with a multilevel fusion and each fusion level consists of one or a series of parallel LE fusions. The structure of PLE fuser is shown schematically in Figure 3.

The PLE fuser contains N fusion levels with N satisfying the inequation $2^{N-1}<L\leq 2^{N}$. In addition, the ith fusion level includes $\left\lfloor L/2^{i}\right\rfloor$ LE fusions and generates $\left\lceil L/2^{i}\right\rceil$ new fused estimates, where notations ⌊∗⌋ and ⌈∗⌉ denote rounding down and rounding up, respectively. The PLE fuser is realized by the following steps:

- Step 1: In the first fusion level, all of the local estimates received from local steady-state Kalman filters are fused in pairs using the LE algorithm. When the number of local estimates is even, we can get $\frac{L}{2}$ new fused estimates; and we can obtain $\frac{L-1}{2}+1$ new fused estimates including an unsettled local estimate when the number of local estimates is odd. Then, the new fused estimates are passed to the next fusion level.
- Step 2: As Step 1, all the estimates received from the upper fusion level are fused in pairs using the LE algorithm, and the obtained new fused estimates are passed to the next fusion level.

⋮

- Step N: There are only two estimates received from the upper fusion level in the fusion level N and the fusion result of them through the LE algorithm is defined as the PLE fuser estimate $N\left({\hat{x}}_{\left(L\right)}^{PLE},P_{\left(L\right)}^{PLE}\right)$.

As shown in Figure 3, we denote the received estimates in the ith fusion level of PLE fuser, respectively, as $E_{1}^{i},E_{2}^{i},\ldots ,E_{Mi}^{i}$ from the left side to the right side, where Mi represents the number of the received estimates in the ith fusion level. Notice that the received estimates in the fusion levels of PLE fuser can be paired by different methods that will lead to different types of PLE fusers with heterogeneous parallel fusion structures. In this paper, we give three estimate pairing methods as follows.

**Method** **1:** In the ith fusion level, the received estimates are paired from $E_{1}^{i}$ to $E_{Mi}^{i}$. If there is an unsettled received estimate in the ith fusion level, it must be $E_{Mi}^{i}$.
**Method** **2:** The fusion levels of this type of PLE fuser alternately pair their received estimates from $E_{1}^{i}$ to $E_{Mi}^{i}$ or from $E_{Mi}^{i}$ to $E_{1}^{i}$. For instance, the local estimates are paired from $E_{1}^{1}$ to $E_{M1}^{1}$ in the fusion level 1, the received estimates in the fusion level 2 are paired from $E_{M2}^{2}$ to $E_{1}^{2}$, and the received estimates in the fusion level 3 are paired from $E_{1}^{3}$ to $E_{M3}^{3}$, etc. If there is an unsettled received estimate in the ith fusion level, it must be $E_{1}^{i}$ or $E_{Mi}^{i}$.
**Method** **3:** In the ith fusion level, the received estimates $E_{Mi}^{i}$ and $E_{1}^{i}$ are grouped into a pair with their fused estimate treated as $E_{1}^{i+1}$ in the next fusion level, and the remaining received estimates are paired from $E_{2}^{i}$ to $E_{Mi-1}^{i}$. If there is an unsettled received estimate in the ith fusion level, it must be $E_{Mi-1}^{i}$.

**Figure 4:**
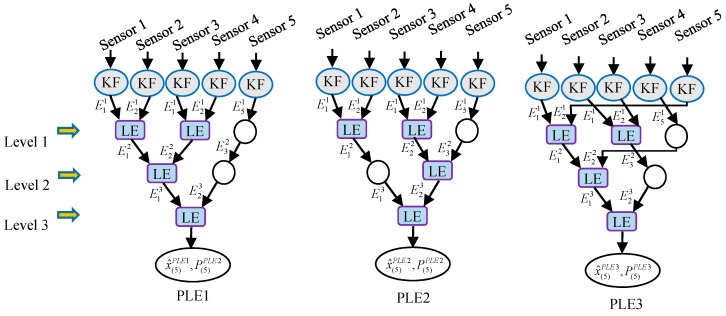
The fusion schemes of PLE1, PLE2 and PLE3 fusers with five sensors.

In the following part of this paper, the notations PLE1, PLE2 and PLE3 denote the PLE fusers, respectively, with estimate pairing Method 1, Method 2 and Method 3. For the multisensor system (1) consisting of five sensors, the fusion schemes of PLE1, PLE2 and PLE3 fusers are shown schematically in Figure 4. In Figure 4, the empty circle at a certain fusion level represents an estimate received from its upper fusion level, and it is the unsettled received estimate, which is directly passed to its next fusion level without fusing with other received estimates.

Similarly to the SLE fuser, the PLE fuser can also be formulated in linear expressions as follows:

(24) $$\begin{array}{l} {\hat{x}}_{\left(L\right)}^{PLE}=\sum_{i=1}^{L}A_{i}^{PLE}{\hat{x}}_{i}\\ P_{\left(L\right)}^{PLE}=\sum_{i=1}^{L}B_{i}^{PLE}P_{i} \end{array},$$

with weighting matrices computed by

(25) $$\begin{array}{c} A_{i}^{PLE}=A_{i(N)}^{LE}\ldots A_{i(2)}^{LE}A_{i(1)}^{LE}\;\;\\ B_{i}^{PLE}=B_{i(N)}^{LE}\ldots B_{i(2)}^{LE}B_{i(1)}^{LE}\; \end{array},$$

where $A_{i(j)}^{LE},B_{i(j)}^{LE}$ indicate the LE fusion weighting matrices corresponding to the LE fusion that the ith local estimate encounters in the jth fusion level in its fusion route; both $A_{i(j)}^{LE}$ and $B_{i(j)}^{LE}$ will be equal to $I_{n}$ if the estimate in the jth fusion level is unsettled in the fusion route.

### 4.3. Properties of Multisensor Largest Ellipsoid Fusers

Comparing (18) to (7), we can find that the state estimation weighting matrices of the LE algorithm will deviate from the optimal weighting matrices, which are the weighting matrices of the optimal distributed Kalman fuser having the same sensors as the LE algorithm, on account of the inaccurate error covariances of local estimates and the presence of unknown cross-covariances. Taking this point into account, the state estimation weighting matrices of SLE fuser and PLE fuser, respectively expressed in (23) and (25) can be rewritten into

(26) $$\begin{array}{l} A_{i}^{SLE}=\left(A_{2/i-1}^{O}+\Delta A_{2/i-1}\right)\prod_{j=i}^{L-1}\left(A_{1/j}^{O}+\Delta A_{1/j}\right)\;\;\;\;\\ A_{i}^{PLE}=\left(A_{i(N)}^{O}+\Delta A_{i(N)}\right)\ldots \left(A_{i(2)}^{O}+\Delta A_{i(2)}\right)\left(A_{i(1)}^{O}+\Delta A_{i(1)}\right)\;\; \end{array},\;\;i=1,2,\ldots ,L,$$

with $A_{2/0}^{O}+\Delta A_{2/0}=I_{n}$. $A_{1/i}^{O},A_{2/i}^{O},\;\;\;i=1,2,\ldots ,L-1$ being the optimal weighting matrices corresponding to the LE fusion in the ith fusion level in the SLE fuser, and $A_{i(j)}^{O}$ being the optimal weighting matrix corresponding to the LE fusion that the ith local estimate encounters in the jth fusion level in its fusion route in the PLE fuser; $\Delta A_{1/i},\Delta A_{2/i},\;\;\;\;i=1,2,\ldots ,L-1$ and $\Delta A_{i(j)}$ are the weighting matrix errors that the state estimation weighting matrices of these LE fusions deviate from their corresponding optimal weighting matrices. Hence, given a multisensor system, the number of the LE fusions that each local estimate encounters in its fusion route and how these numbers differ from each other will affect the weight assignments for the local estimates in the fuser, which implies that the fuser structure has a significant influence on the characteristic and performance of the fuser. From Figure 2 and Figure 3, we see that the number of the LE fusions that each local estimate encounters in its fusion route can be different in the SLE fuser and the PLE fusers based on different estimate pairing methods. In order to give a further analysis of the features of SLE fuser and PLE fuser for multisensor system (1), here we define two fusion performance assessment parameters as Fusion Distance and Fusion Index.

**Definition** **1.** *The Fusion Distance* $D_{\left(i\right)}^{\left(j\right)}$ *indicates the number of the LE fusions that the* i*th local estimate encounters in its fusion route in fuser* j *(SLE, PLE1, PLE2 or PLE3).*

**Remark** **1.** *When* L≥2*, we have* $\max(D_{\left(i\right)}^{\left(SLE\right)})=L-1$ *and* $\mathrm{minx}(D_{\left(i\right)}^{\left(SLE\right)})=1$*. For any certain* N≥1*, we have* $\max(D_{\left(i\right)}^{\left(PLE1\right)})=\max(D_{\left(i\right)}^{\left(PLE2\right)})=\max(D_{\left(i\right)}^{\left(PLE3\right)})=N$*,* $\min(D_{\left(i\right)}^{\left(PLE1\right)})=1$*,* $\min(D_{\left(i\right)}^{\left(PLE2\right)})=N-\left\lceil \frac{N-1}{2}\right\rceil$*, and* $\min(D_{\left(i\right)}^{\left(PLE3\right)})=\max\left(N-1,1\right)$.

**Definition** **2.** *The Fusion Index* $F^{\left(j\right)}$ *shows to what extent the Fusion Distances of all the local estimates differ from each other in fuser* j *(SLE, PLE1, PLE2 or PLE3). It is defined as*

(27) $$F^{\left(j\right)}=\max\left(\underset{i=1,2,\ldots ,L}{D_{\left(i\right)}^{\left(j\right)}}\right)-\min\left(\underset{i=1,2,\ldots ,L}{D_{\left(i\right)}^{\left(j\right)}}\right).$$

**Remark** **2.** *When*  L≥2*, we have* $F^{\left(SLE\right)}=L-2$*. For any certain* N≥1*, we have* $\max\left(F^{\left(PLE1\right)}\right)=N-1$*,* $\max\left(F^{\left(PLE2\right)}\right)=\left\lceil \frac{N-1}{2}\right\rceil$*,* $\max\left(F^{\left(PLE3\right)}\right)=\min\left(N-1,1\right)$.

Because the fused error covariance ellipsoid of LE algorithm is contained within the intersection region of the covariance ellipsoids of local estimates, we can easily come to the conclusion that $P_{\left(L\right)}^{SLE}\leq P_{i},P_{\left(L\right)}^{PLE}\leq P_{i},\;\;\;\;\;i=1,2,\ldots ,L$. However, as we can see from (23) and (25), the error covariance estimation weighting matrices of SLE fuser and different PLE fusers are varied with the structure, hence the fused error covariances of these fusers are generally different from each other. For multisensor system (1), when adding a new sensor to the system, we apparently have $P_{\left(L+1\right)}^{SLE}\leq P_{\left(L\right)}^{SLE}$. However, the situation of the PLE fuser is more complicated, but it is obvious that if the existing fusion structure of a PLE fuser is not affected by the new sensor, then the one PLE fuser will embrace $P_{\left(L+1\right)}^{PLE}\leq P_{\left(L\right)}^{PLE}$, such as the PLE1 fuser. The fused accuracies of SLE fuser and PLE fuser with such property will become higher and higher as the number of fused sensors increases. In the sense that both ${\hat{x}}_{\left(L\right)}^{O}$, ${\hat{x}}_{\left(L\right)}^{SLE}$ and ${\hat{x}}_{\left(L\right)}^{PLE}$ are the linear unbiased estimates of state x and ${\hat{x}}_{\left(L\right)}^{O}$ is the LUMV estimate, we have $P_{\left(L\right)}^{O}\leq {\bar{P}}_{\left(L\right)}^{SLE}$ and $P_{\left(L\right)}^{O}\leq {\bar{P}}_{\left(L\right)}^{PLE}$, where ${\bar{P}}_{\left(L\right)}^{SLE}=E\left[\left({\hat{x}}_{\left(L\right)}^{SLE}-x\right){\left({\hat{x}}_{\left(L\right)}^{SLE}-x\right)}^{T}\right]$ and ${\bar{P}}_{\left(L\right)}^{PLE}=E\left[\left({\hat{x}}_{\left(L\right)}^{PLE}-x\right){\left({\hat{x}}_{\left(L\right)}^{PLE}-x\right)}^{T}\right]$ are the actual fused error covariances of the SLE fuser and PLE fuser, respectively. In addition, we can achieve the upper bounds for the actual fused error covariances of the SLE fuser and PLE fuser irrespective of the cross-covariances. For multisensor system (1), according to [24], for any factors $\infty \geq \rho_{i}\geq 1,\;i=1,2,\ldots ,L$, when

(28) $$\sum_{i=1}^{L}\frac{1}{\rho_{i}}=1,$$

we have

(29) $$\Sigma \leq \left[\begin{array}{ccc} \rho_{1}P_{1} & 0 & 0\\ 0 & \ddots & 0\\ 0 & 0 & \rho_{L}P_{L} \end{array}\right],$$

For the SLE fuser and PLE fuser, we obtain

(30) $$\begin{array}{ll} {\bar{P}}_{\left(L\right)}^{k} & =\left[A_{1}^{k},\ldots ,A_{L}^{k}\right]\Sigma {\left[A_{1}^{k},\ldots ,A_{L}^{k}\right]}^{T}\\ & \leq \left[A_{1}^{k},\ldots ,A_{L}^{k}\right]\left[\begin{array}{ccc} \rho_{1}P_{1} & 0 & 0\\ 0 & \ddots & 0\\ 0 & 0 & \rho_{L}P_{L} \end{array}\right]{\left[A_{1}^{k},\ldots ,A_{L}^{k}\right]}^{T}\\ & =\sum_{i=1}^{L}\rho_{i}A_{i}^{k}P_{i}{\left(A_{i}^{k}\right)}^{T},\;k\in \left(SLE,PLE\right) \end{array},$$

By taking the minimization of error covariance trace as the optimization target, we get the optimization model as

(31) $$\begin{array}{l} \min\left\{\sum_{i=1}^{L}\rho_{i}\mathrm{tr}\left[A_{i}^{k}P_{i}{\left(A_{i}^{k}\right)}^{T}\right]\right\}\\ s.t.\;\sum_{i=1}^{L}\frac{1}{\rho_{i}}=1 \end{array}.$$

Applying the Lagrange multiplier method, we introduce the Lagrange function defined by

(32) $$f\left(\rho_{1},\ldots ,\rho_{L},\lambda \right)=\sum_{i=1}^{L}\rho_{i}\mathrm{tr}\left[A_{i}^{k}P_{i}{\left(A_{i}^{k}\right)}^{T}\right]+\lambda \left(\sum_{i=1}^{L}\frac{1}{\rho_{i}}-1\right),$$

where λ is the Lagrange multiplier. Then, we can achieve

(33) $$\rho_{i}=\frac{\sum_{j=1}^{L}\sqrt{\mathrm{tr}\left[A_{j}^{k}P_{j}{\left(A_{j}^{k}\right)}^{T}\right]}}{\sqrt{\mathrm{tr}\left[A_{i}^{k}P_{i}{\left(A_{i}^{k}\right)}^{T}\right]}}.$$

Replacing (33) into (30), we obtain

(34) $${\bar{P}}_{\left(L\right)}^{k}\leq \sum_{i=1}^{L}\frac{\sum_{j=1}^{L}\sqrt{\mathrm{tr}\left[A_{j}^{k}P_{j}{\left(A_{j}^{k}\right)}^{T}\right]}}{\sqrt{\mathrm{tr}\left[A_{i}^{k}P_{i}{\left(A_{i}^{k}\right)}^{T}\right]}}A_{i}^{k}P_{i}{\left(A_{i}^{k}\right)}^{T},\;k\in \left(SLE,PLE\right).$$

## 5. Simulations and Analysis

### 5.1. Simulations

Consider a dynamic example of the multisensor system (1) with five sensors as

(35) $$\begin{array}{l} x\left(t+1\right)=\left[\begin{array}{cc} 1 & T\\ 0 & 1 \end{array}\right]x\left(t\right)+\left[\begin{array}{c} 0.5T^{2}\\ T \end{array}\right]w\left(t\right)\\ y_{i}\left(t\right)=H_{i}x\left(t\right)+v_{i}\left(t\right)，\;i=1,2,\ldots ,5 \end{array},$$

where T=0.5 is the sample period, t=1,2,…,300 is the discrete time (step), $x_{0}={\left[10,2\right]}^{T}$ is the initial state, Q=2, and

(36) $$\begin{array}{l} H_{1}=I_{2},R_{1}=\mathrm{diag}\left(7.0,0.22\right)\\ H_{2}=I_{2},R_{2}=\mathrm{diag}\left(2.85,0.3\right)\\ H_{3}=I_{2},R_{3}=\mathrm{diag}\left(1.3,1.5\right)\\ H_{4}=I_{2},R_{4}=\mathrm{diag}\left(0.55,3.1\right)\\ H_{5}=\left[1,0\right],R_{5}=0.6 \end{array}.$$

**Figure 5:**
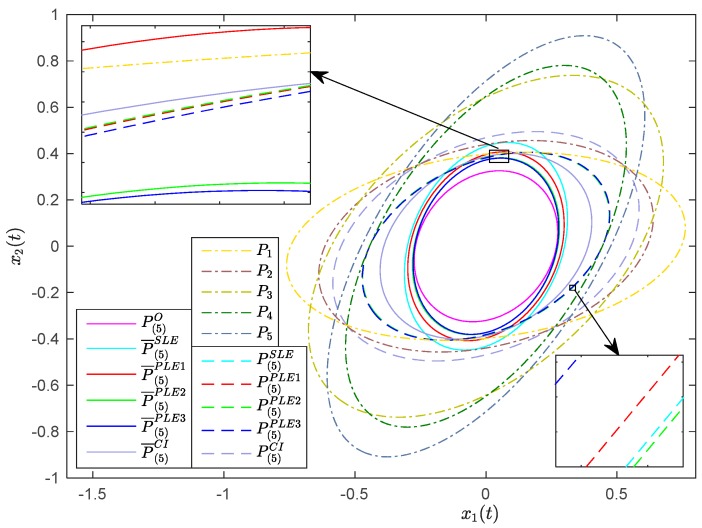
The error covariance ellipses of local and fused estimates.

The local steady-state Kalman estimates, local filtering error covariances and cross-covariances can be obtained according to (2)–(5), and then the fused estimates of the optimal distributed Kalman fuser weighted by matrices, the CI algorithm, the SLE fuser and PLE fusers can be computed. The fusion schemes of PLE1, PLE2 and PLE3 fusers are shown in Figure 4. The traces of the theoretical error covariance matrices of local and fused estimates are listed in Table 1, and their corresponding covariance ellipses are illustrated in Figure 5. In Figure 5, the center of each covariance ellipse is x(t), and the *x*-axis and the *y*-axis indicate how far the covariance ellipses extend in the directions of the first and second dimension of the state from the center, respectively.

From Table 1, we see that both $\mathrm{tr}\left({\bar{P}}_{\left(5\right)}^{SLE}\right)$ and $\mathrm{tr}\left({\bar{P}}_{\left(5\right)}^{PLEi}\right),\;i=1,2,3$ are greater than but close to $\mathrm{tr}\left(P_{\left(5\right)}^{O}\right)$, which imply that the actual fused accuracies of SLE fuser and PLE fusers are close to that of the optimal fuser. The $\mathrm{tr}\left(P_{\left(5\right)}^{SLE}\right)$ and $\mathrm{tr}\left(P_{\left(5\right)}^{PLEi}\right),\;i=1,2,3$ are almost the same and obviously less than $\mathrm{tr}\left(P_{\left(5\right)}^{CI}\right)$ and $\mathrm{tr}\left(P_{i}\right),\;i=1,2,\ldots ,5$, which mean that the fused accuracies of SLE fuser and PLE fusers are approximately equal and higher than those of the CI algorithm and each local estimate. Note that, although the $\mathrm{tr}\left({\bar{P}}_{\left(5\right)}^{SLE}\right)$ and $\mathrm{tr}\left({\bar{P}}_{\left(5\right)}^{PLEi}\right),\;i=1,2,3$ are, respectively, less than $\mathrm{tr}\left(P_{\left(5\right)}^{SLE}\right)$ and $\mathrm{tr}\left(P_{\left(5\right)}^{PLEi}\right),\;i=1,2,3$, it can not be concluded that the SLE fuser and PLE fusers are consistent due to fact that the LE algorithm can not guarantee its consistency. Correspondingly, in Figure 5, the covariance ellipse of $P_{\left(5\right)}^{O}$ is enclosed in the covariance ellipses of ${\bar{P}}_{\left(5\right)}^{SLE}$ and ${\bar{P}}_{\left(5\right)}^{PLEi},\;i=1,2,3$, which indicates $P_{\left(5\right)}^{O}\leq {\bar{P}}_{\left(5\right)}^{SLE}$ and $P_{\left(5\right)}^{O}\leq {\bar{P}}_{\left(5\right)}^{PLEi},\;i=1,2,3$. The covariance ellipses of $P_{\left(5\right)}^{SLE}$ and $P_{\left(5\right)}^{PLEi},\;i=1,2,3$ are enclosed in the covariance ellipses of $P_{\left(5\right)}^{CI}$ and $P_{i},\;i=1,2,\ldots ,5$, which indicate $P_{\left(5\right)}^{SLE}\leq P_{i},\;i=1,2,\ldots ,5$, $P_{\left(5\right)}^{SLE}\leq P_{\left(5\right)}^{CI}$, $P_{\left(5\right)}^{PLEj}\leq P_{i},\;i=1,2,\ldots ,5;j=1,2,3$ and $P_{\left(5\right)}^{PLEj}\leq P_{\left(5\right)}^{CI},\;j=1,2,3$. The covariance ellipses of $P_{\left(5\right)}^{SLE}$ and $P_{\left(5\right)}^{PLEi},\;i=1,2,3$ are almost overlapping, which implies that the SLE fuser and PLE fusers each obtain a similar fused error covariance. However, the covariance ellipse of ${\bar{P}}_{\left(5\right)}^{SLE}$ is not enclosed in the covariance ellipses of $P_{\left(5\right)}^{SLE}$ and $P_{1}$, which means that ${\bar{P}}_{\left(5\right)}^{SLE}≰P_{\left(5\right)}^{SLE}$ and ${\bar{P}}_{\left(5\right)}^{SLE}≰P_{1}$; and the covariance ellipse of ${\bar{P}}_{\left(5\right)}^{PLE1}$ is not enclosed in the covariance ellipses of $P_{\left(5\right)}^{PLE1}$ and $P_{1}$, which means ${\bar{P}}_{\left(5\right)}^{PLE1}≰P_{\left(5\right)}^{PLE1}$ and ${\bar{P}}_{\left(5\right)}^{PLE1}≰P_{1}$; thus, the SLE fuser and PLE1 fuser are inconsistent in this example. While the covariance ellipses of ${\bar{P}}_{\left(5\right)}^{PLE2}$ and ${\bar{P}}_{\left(5\right)}^{PLE3}$ are, respectively, enclosed in the covariance ellipses of $P_{\left(5\right)}^{PLE2}$ and $P_{\left(5\right)}^{PLE3}$, which mean ${\bar{P}}_{\left(5\right)}^{PLE2}\leq P_{\left(5\right)}^{PLE2}$ and ${\bar{P}}_{\left(5\right)}^{PLE3}\leq P_{\left(5\right)}^{PLE3}$, the PLE2 and PLE3 fusers are consistent here.

In order to verify the above theoretical results on the accuracy relation, the Monte Carlo method is applied to compute mean square error (MSE). The MSE value at time t for local or fused estimate ${\hat{x}}^{i}$ with error covariance $P^{i}$ is defined as

(37) $$MSE_{N_{run}}^{i}\left(t\right)=\frac{1}{N_{run}}\sum_{j=1}^{N_{run}}{\left[{\hat{x}}_{j}^{i}\left(t|t\right)-x_{j}\left(t\right)\right]}^{T}\left[{\hat{x}}_{j}^{i}\left(t|t\right)-x_{j}\left(t\right)\right],$$

where $N_{run}$ is the number of Monte Carlo runs, ${\hat{x}}_{j}^{i}\left(t|t\right)$ and $x_{j}\left(t\right)$ denote the jth realization of ${\hat{x}}^{i}\left(t|t\right)$ and x(t), respectively. Because

(38) $$\mathrm{tr}\left(P^{i}\right)=\mathrm{tr}\left\{E\left[\left({\hat{x}}^{i}-x\right){\left({\hat{x}}^{i}-x\right)}^{T}\right]\right\}=E\left[{\left({\hat{x}}^{i}-x\right)}^{T}\left({\hat{x}}^{i}-x\right)\right],$$

according to the ergodicity [25], we have

(39) $$MSE_{N_{run}}^{i}\left(t\right)=\mathrm{tr}\left(P^{i}\right),\;t\to \infty ,N_{run}\to \infty .$$

**Figure 6:**
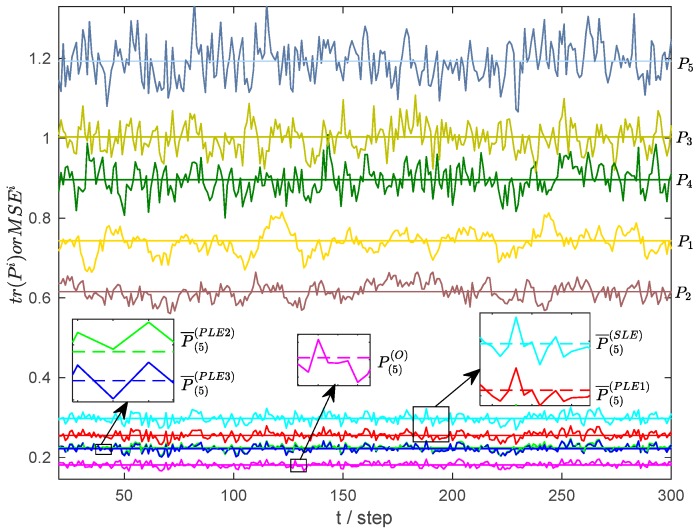
The tr(i) and $MSE^{i}$ of local and fused estimates.

For the dynamic example (35)–(36), 1000 Monte Carlo runs are performed, and the statistical results of local estimates and the fused estimates of optimal fuser, SLE fuser and PLE fusers in the Monte Carlo simulation are shown in Figure 6.

In Figure 6, the straight lines and dashed lines denote $\mathrm{tr}\left(P^{i}\right)$, and the solid curves denote $MSE_{N_{run}}^{i}$; $P^{i}$ represents $P_{i},\;i=1,2,\ldots ,5$, $P_{\left(5\right)}^{O}$, ${\bar{P}}_{\left(5\right)}^{SLE}$ and ${\bar{P}}_{\left(5\right)}^{SLE},\;i=1,2,3$ , while $MSE_{N_{run}}^{i}$, denotes their corresponding MSE values. From Figure 6, we know that the $MSE_{N_{run}}^{i}$ fluctuates around $\mathrm{tr}\left(P^{i}\right)$, which is consistent with (39); and the statistical accuracy relations of local and fused estimates indicated by $MSE_{N_{run}}^{i}$ are coincident with the theoretical results shown in Table 1.

**Figure 7:**
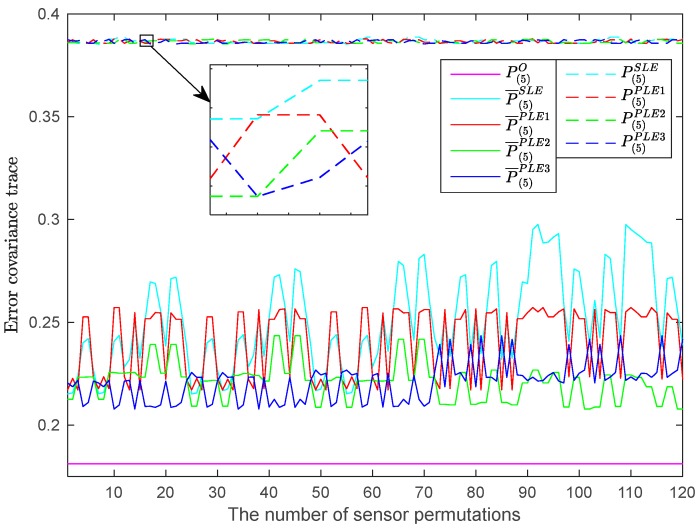
The fuser accuracies with respect to different orders of five sensors.

As shown in Figure 2 and Figure 3, both the SLE fuser and PLE fuser schemes will vary as long as the sensor order varies. To explore how the accuracies of SLE fuser and the PLE fusers based on different estimate pairing methods are related to the sensor orders, all the sensor permutations are considered and simulated. The accuracies of SLE fuser and PLE fusers with respect to different sensor orders are given in Figure 7. In Figure 7, the *x*-axis represents all of the permutations of five used sensors, 120 in total; the *y*-axis represents the traces of $P_{\left(5\right)}^{O}$, ${\bar{P}}_{\left(5\right)}^{SLE}$, ${\bar{P}}_{\left(5\right)}^{PLEi},\;i=1,2,3$ and $P_{\left(5\right)}^{SLE}$, $P_{\left(5\right)}^{PLEi},\;i=1,2,3$.

Figure 7 shows that, for all possible sensor orders, the fused accuracies of SLE fuser and PLE fusers are almost the same, while their actual fused accuracies are more different, which mean that the fused accuracies of SLE fuser and PLE fusers are less affected by the sensor orders, but their actual fused accuracies are greatly influenced. In order to strengthen the information shown in Figure 7, another four sensors are added to systems (35) and (36), and they are given as

(40) $$\begin{array}{l} H_{6}=I_{2},R_{6}=\mathrm{diag}\left(2.1,2.06\right)\\ H_{7}=I_{2},R_{7}=\mathrm{diag}\left(1.1,7.56\right)\\ H_{8}=I_{2},R_{8}=\mathrm{diag}\left(16.6,0.15\right)\\ H_{9}=I_{2},R_{9}=\mathrm{diag}\left(0.9,23.0\right) \end{array}.$$

In addition, the traces of the theoretical error covariance matrices of their local steady-state Kalman estimates are shown in Table 2.

**Figure 8:**
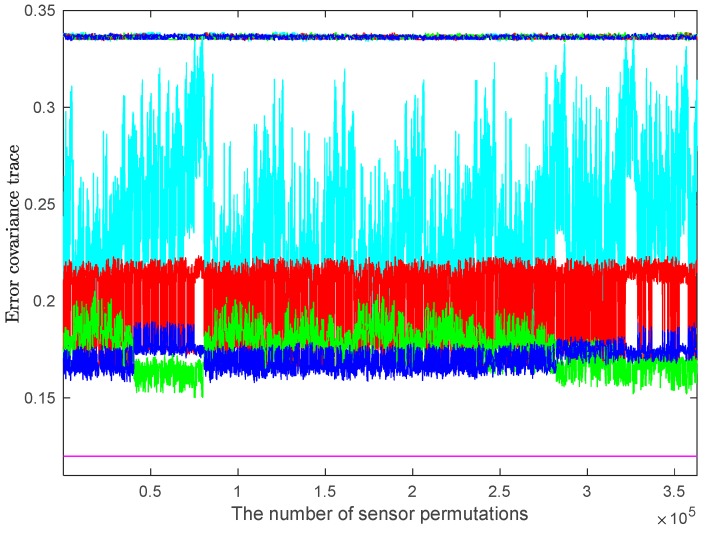
The fuser accuracies with respect to different orders of nine sensors.

The accuracies of SLE fuser and PLE fusers with respect to different sensor orders for the expanded multisensor system with nine sensors are shown in Figure 8. In Figure 8, the *x*-axis represents all of the permutations of nine used sensors, 362,880 in total; and the legends of this figure are the same as those in Figure 7.

From Figure 7 and Figure 8, it can be seen that with a certain number of sensors, no matter how the sensors are ordered, the $\mathrm{tr}\left(P^{SLE}\right)$ and $\mathrm{tr}\left(P^{PLEi}\right),\;i=1,2,3$ are almost equal, which mean that the fused accuracies of SLE fuser and PLE fusers are almost equivalent and are insensitive to the sensor orders. When the multisensor system only has a few sensors, the actual fused accuracies of SLE fuser and PLE fusers perform approximately and have similar sensitivity to sensor orders. However, as the number of sensors increases, the differences among them are becoming increasingly significant. From Figure 8, we see that the $\mathrm{tr}\left({\bar{P}}^{SLE}\right)$ fluctuates most drastically and is generally greater than $\mathrm{tr}\left({\bar{P}}^{PLEi}\right),\;i=1,2,3$, which means that the actual fused accuracy of SLE fuser is poorer and more sensitive to the sensor orders than these of PLE fusers. Whether in actual fused accuracy or in sensitivity to the sensor orders, the PLE2 and PLE3 fusers perform better than the PLE1 fuser, and the PLE3 fuser generally has the best performance.

**Figure 9:**
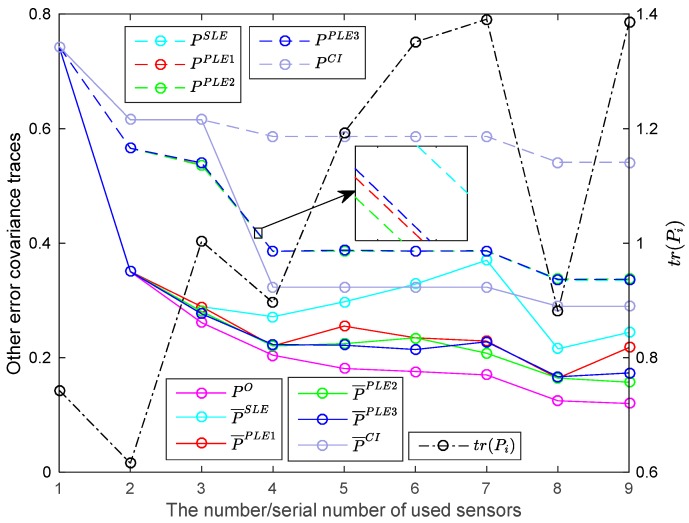
The fuser accuracies with respect to different numbers of used sensors.

In practice, the number of used sensors may vary in different periods. For the subsystems individually with sensors 1∼i, i=1,2,…,9, the accuracies of the SLE fuser and PLE fusers with sensors fused in normal order are presented in Figure 9. In Figure 9, the number i on the *x*-axis not only represents the ith subsystem using the sensors 1∼i, but also represents the ith sensor. From Figure 9, we know that the actual fused accuracy of SLE fuser is less robust to the accuracy of the newly added sensor than these of PLE fusers; and, compared to the PLE1 fuser, the actual fused accuracies of PLE2 and PLE3 fusers perform more robustly and they tend to become higher as the number of sensors increases. Furthermore, the accuracies of PLE fusers are significantly better than that of the CI algorithm.

**Figure 10:**
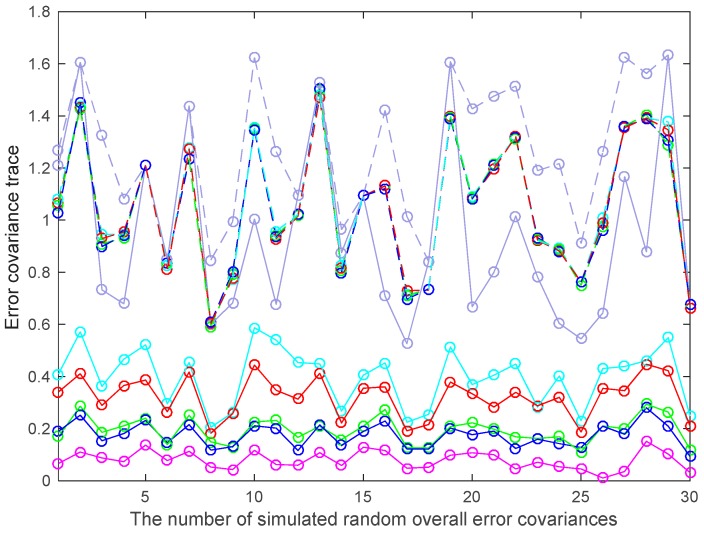
The fuser accuracies with respect to different random overall error covariance matrices.

In the simulation cases above, we have specified the characteristics of the sensors. Without such specifications, we give a further study on the performances of the SLE fuser and PLE fusers in the multisensor systems with arbitrary overall error covariances. Because the overall error covariance Σ of the multisensor system (1) is a real symmetric positive definite matrix, it has diagonal decomposition as $\Sigma \;=\;\Theta \Xi \Theta^{T}$, where Θ is an orthogonal matrix, Ξ is a diagonal matrix. Θ and Ξ can be randomly created using Matlab (R2015b, MathWorks, Natick, MA, US) functions, such as ‘orth’, ‘diag’ and ‘randn’, etc. For the multisensor system consisting of nine two-dimensional sensors, 30 random overall error covariance matrices are simulated with each element of their Ξ selected from the positive samples of a random variable, which is of standard normal distribution, and the fused results of SLE fuser and PLE fusers are shown in Figure 10. In Figure 10, the *x*-axis represents the 30 simulated random overall error covariances, and the legends of this figure are the same as these in Figure 9. As shown in Figure 10, for most of the simulated overall error covariance matrices, the multisensor LE fusers sorted in descending order of actual fused accuracy are PLE3, PLE2, PLE1 and the SLE fuser, and they all have higher accuracies than the CI algorithm.

**Figure 11:**
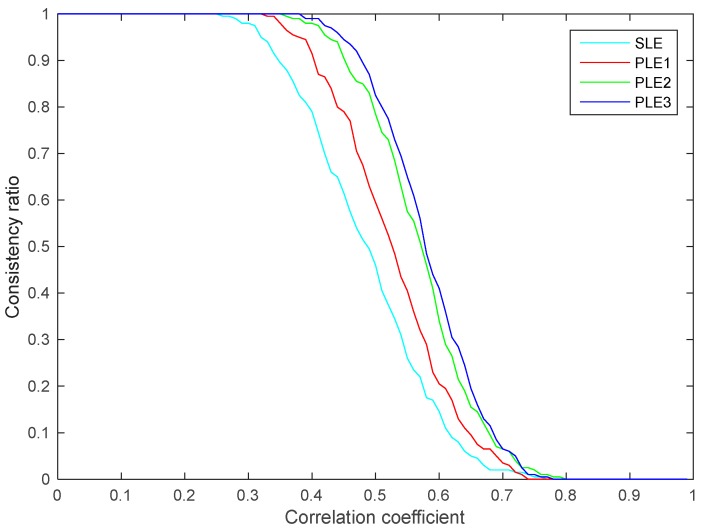
The consistency ratios with respect to different correlation coefficients.

For any two local estimates ${\hat{x}}_{i}$ and ${\hat{x}}_{j}$ in the multisensor system, the correlation property of them can be measured by the following correlation model [11]:

(41) $$P_{ij}=\gamma J_{i}J_{j}^{T},$$

where γ∈[0,1) is the correlation coefficient between ${\hat{x}}_{i}$ and ${\hat{x}}_{j}$; $J_{k},\;k=i,j$ is the Cholesky decomposition of $P_{k}$ satisfying $J_{k}J_{k}^{T}=P_{k}$. As described above, the inconsistency of the LE algorithm is resulted from the unknown cross-covariances among local estimates. In order to investigate how the consistencies of SLE fuser and PLE fusers are related to the unknown cross-covariances, the multisensor systems consisting of nine two-dimensional sensors under different correlation coefficients are studied. Meanwhile, in order to cover the main range of the correlation coefficient, in this case, each studied correlation coefficient is given by $\gamma_{i}=0.01n_{i}$, where the integer $n_{i}\in \left[0,99\right]$ is the serial number of the correlation coefficients. For each performed correlation coefficient $\gamma_{i}$, 200 random overall error covariance matrices of the multisensor system are firstly created, and then in each overall error covariance matrix, the cross-covariance between any two local estimates is replaced by the new cross-covariance obtained using (41). The consistency ratios of the number of consistent fused results to the total number of fused results for SLE fuser and PLE fusers with respect to different correlation coefficients are shown in Figure 11.

From Figure 11, we know that the presented multisensor LE fusers are inconsistent when the local estimates are strongly correlated but are consistent when the local estimates are weakly correlated. Under a moderate correlation level, the multisensor LE fusers sorted in a descending order of the consistency ratio are PLE3, PLE2, PLE1 and SLE fusers. For certain moderate $\gamma_{i}$, compared to the SLE fuser, the PLE fusers significantly improve the consistency performance.

In summary, according to above simulation results, whether in the actual fused accuracy as well as its sensitivity to the sensor orders and its robustness to the accuracy of a newly added sensor, or in consistency and in computation efficiency, the PLE fusers have better performances than SLE fuser, and PLE3 fuser outperforms PLE2 fuser, which performs better than the PLE1 fuser.

### 5.2. Analysis

In order to obtain a fused estimate with high actual fused accuracy in a multisensor LE fuser, the weights of the state estimation weighting matrices should be assigned in accordance with the accuracies of sensors. As shown in (23) and (25), both $A_{i}^{SLE}$ in SLE fuser and $A_{i}^{PLE}$ in PLE fuser are the multiplication results of all of the LE fusion state estimation weighting matrices that the ith local estimate encounters in its fusion route. From (18), we have $A_{1}^{LE}<I_{n}$ and $A_{2}^{LE}<I_{n}$, the multiplication effect of multiple $A_{1}^{LE}$ and (or) $A_{2}^{LE}$ implies that the more LE fusions one local estimate encounters in its fusion route in the SLE fuser or PLE fuser, the less weight its weighting matrix $A_{i}^{SLE}$ or $A_{i}^{PLE}$ will tend to be. Since the number of the LE fusions that each local estimate encounters in its fusion route is affected by the fuser structure, the weights of the weighting matrices not only depend on the accuracies of sensors, but also are influenced by fuser structure. In addition, according to (26), the number of LE fusions that each local estimate encounters in its fusion route also affects the weighting matrix deviations from the optimal weighting matrices.

Therefore, longer Fusion Distance means less weight and greater deviation; the Fusion Distance differences in the fuser affect the balances of weight assignment and deviation among local estimates and thus have a significant influence on the fuser’s actual fused accuracy performance. Owing to the sequential structure, the Fusion Distances of each local estimate in the SLE fuser are remarkably different from each other, which leads to the fuser structure severely degrading the dependency of the actual fused accuracy on the accuracies of sensors. Comparatively, the differences of the local estimate Fusion Distances in PLE fuser are significantly reduced, which means that the actual fused accuracy of PLE fuser is more dependent on the accuracies of sensors and hence is better than that of the SLE fuser. The Fusion Index of the multisensor LE fuser not only represents the fuser’s actual fused accuracy, but also indicates the sensitivity of the actual fused accuracy to the sensor orders and the robustness of the actual fused accuracy to the accuracy of the newly added sensor. A smaller Fusion Index means that the fuser has better performances in these aspects. In the simulation examples, when the multisensor system has five sensors, we have $F^{\left(SLE\right)}=3$, $F^{\left(PLE1\right)}=2$, $F^{\left(PLE2\right)}=F^{\left(PLE3\right)}=1$, the PLE3, PLE2 fusers have the same level of performance in actual fused accuracy, sensitivity and robustness, and perform better than the SLE fuser and PLE1 fuser. When the multisensor system has nine sensors, we have $F^{\left(SLE\right)}=7$, $F^{\left(PLE1\right)}=3$, $F^{\left(PLE2\right)}=2$, $F^{\left(PLE3\right)}=1$, and the performance differences among SLE, PLE1, PLE2, PLE3 fusers become more significant. As the fused error covariances of the SLE fuser and PLE fuser are almost the same, then, for the same multisensor system, the fuser that has higher actual fused accuracy will have less possibility to become inconsistent. Accordingly, compared to the SLE fuser, the PLE fuser has better consistency performance, and the PLE2 fuser has worse performance than the PLE3 fuser but has better performance than the PLE2 fuser.

Apparently, compared to the SLE fuser, another one of the advantages of the PLE fuser is that the PLE fuser can make full use of the multiprocessor to fuse multiple sensors in parallel and more efficiently. Because each LE fusion can reduce the estimates to be fused by one in the SLE fuser and PLE fuser, it can be concluded that both the SLE fuser and the PLE fuser for the multisensor system (1) contain L−1 LE fusions, and thus they have the same computation complexity. However, if multiprocessor parallel operation is used in data processing, their time complexity is proportional to their number of fusion levels. Therefore, the PLE fuser mostly outperforms the SLE fuser in computation efficiency. When the sensors are in a clustering distribution, parallel structure can also cut down the communication requirements of sensor networks.

## 6. Conclusions

For the multisensor system with unknown cross-covariances, this paper proposes a largest ellipsoid fusion Kalman filtering with parallel structure and gives three different estimate pairing methods to construct the parallel fusion structure. Two fusion performance assessment parameters of Fusion Distance and Fusion Index are defined, and the attributes of the SLE fuser and PLE fusers in Fusion Distance, Fusion Index and accuracy relation are given. Verified with examples, if a local estimate has a longer Fusion Distance, its weighting matrix will tend to be lighter in weight and of greater deviation. The Fusion Index reflects the influence of fusion structure on fusion performance and indicates a fuser’s actual fused accuracy performance. A smaller Fusion Index implies that the actual fused accuracy of the fuser is generally higher, and is less sensitive to the sensor orders and more robust to the accuracy of newly added sensors. The presented multisensor LE fusers can achieve consistent fused results when the local estimates are weakly correlated but become inconsistent when the local estimates are strongly correlated; under such strong correlations, the upper bounds of actual fused error covariances of the presented multisensor LE fusers can be obtained by the provided formula to limit the uncertainties of the fused results. Compared to SLE fusers, the proposed PLE fusers not only can operate in parallel and more efficiently, but also get better performances in regards to accuracy and consistency.

## Figures and Tables

**Table 1:** The traces of theoretical error covariance matrices.

$\mathrm{tr}\left(P_{1}\right)$	$\mathrm{tr}\left(P_{2}\right)$	$\mathrm{tr}\left(P_{3}\right)$	$\mathrm{tr}\left(P_{4}\right)$	$\mathrm{tr}\left(P_{5}\right)$	$\mathrm{tr}\left(P_{\left(5\right)}^{O}\right)$	$\mathrm{tr}\left({\bar{P}}_{\left(5\right)}^{CI}\right)$	$\mathrm{tr}\left(P_{\left(5\right)}^{CI}\right)$
0.7433	0.6155	1.0032	0.8962	1.1932	0.1812	0.3233	0.5863
$\mathrm{tr}\left({\bar{P}}_{\left(5\right)}^{SLE}\right)$	$\mathrm{tr}\left(P_{\left(5\right)}^{SLE}\right)$	$\mathrm{tr}\left({\bar{P}}_{\left(5\right)}^{PLE1}\right)$	$\mathrm{tr}\left(P_{\left(5\right)}^{PLE1}\right)$	$\mathrm{tr}\left({\bar{P}}_{\left(5\right)}^{PLE2}\right)$	$\mathrm{tr}\left(P_{\left(5\right)}^{PLE2}\right)$	$\mathrm{tr}\left({\bar{P}}_{\left(5\right)}^{PLE3}\right)$	$\mathrm{tr}\left(P_{\left(5\right)}^{PLE3}\right)$
0.2976	0.3861	0.2550	0.3860	0.2244	0.3861	0.2217	0.3876

**Table 2:** The traces of the theoretical error covariance matrices of added local estimates.

$\mathrm{tr}\left(P_{6}\right)$	$\mathrm{tr}\left(P_{7}\right)$	$\mathrm{tr}\left(P_{8}\right)$	$\mathrm{tr}\left(P_{9}\right)$
1.3512	1.3910	0.8807	1.3865
